# Visual dominance of the congruency sequence effect in a cross-modal context

**DOI:** 10.3389/fpsyg.2024.1504068

**Published:** 2024-12-18

**Authors:** Xiaoyu Tang, Xi Zhang, Tingting Wang, Hongtao Yu, Aijun Wang, Ming Zhang

**Affiliations:** ^1^School of Psychology, Liaoning Collaborative Innovation Center of Children and Adolescents Healthy Personality Assessment and Cultivation, Liaoning Normal University, Dalian, China; ^2^Department of Psychology, Soochow University, Suzhou, China; ^3^Department of Psychology, Suzhou University of Science and Technology, Suzhou, China; ^4^Faculty of Interdisciplinary Science and Engineering in Health Systems, Okayama University, Okayama, Japan

**Keywords:** cognitive control, congruency sequence effect, cross-modal, conflict adaptation, visual dominance

## Abstract

The congruency sequence effect (CSE) refers to the reduction in the congruency effect in the current trial after an incongruent trial compared with a congruent trial. Although previous studies widely suggested that CSE was observed only in the modality repeat condition, few studies have reported that CSE could also appear in the modality switch condition. However, it remains unclear whether these conflicting findings were caused by partial repetition effects under modality transition conditions. To address this issue, Experiment 1 controlled for partial repetition effects by ensuring that the modality relationships in both the repetition and switch conditions were either fully congruent or incongruent. The results revealed significant CSE only under the modality repetition condition. In particular, a larger CSE was observed in visual–auditory (VA) repetition than in auditory–visual (AV) repetition, indicating that modality asymmetry might affect the CSE by inducing the priming effect. Thus, Experiment 2 concurrently presented visual and auditory stimuli to eliminate priming effects and further validated CSE differences between auditory and visual modalities. The results revealed that the CSE was significantly greater under the VA condition than under the AV condition and confirmed that the visual modality played a dominant role in the CSE, as visual information is prioritized in processing and ultimately reduces the congruency effect in the next trial. Overall, the present study provides evidence for the specificity of CSE under modality repetition conditions by excluding partial repetition effects and further underscores the critical role of visual dominance in cross-modal CSE.

## 1 Introduction

Human performance on a current task is often influenced by prior experience. In prime-probe tasks, for example, participants are asked to identify the probe color while ignoring the prime color (Weissman et al., 2016; Kelber et al., 2023). When the prime and probe colors conflict, responses are typically slower and less accurate than when they match (Botvinick, 2007; Braem et al., 2014; Atalay and Inan, 2017). In particular, such a congruency effect is smaller when the previous trial was consecutively incongruent than when it was congruent (Congruency Sequence Effect, termed CSE; see Grant and Weissman, 2019; Schlaghecken and Maylor, 2020). The CSE has been widely suggested to reflect the control adjustments that modulate response activation after the prime appears but before the probe is presented (Spapé and Hommel, 2008; Kelber et al., 2024). Importantly, evidence has shown that CSE can be modulated by transitions in context features (e.g., modality relationships) between two consecutive trials in prime-probe task (Bräutigam, 2024; Gratton et al., 1992; Kelber et al., 2023).

Evidence widely suggests that CSE is domain specific, occurring when two consecutive trials have repetition features but disappearing in trials with different features (Frings and Spence, 2010; Grant et al., 2020; Yang et al., 2021). For example, by using the prime-probe task, Kelber et al. (2023) investigated the effect of context repetition on CSE by manipulating the stimulus modality, such as spoken color words or color patches. The study revealed significant CSE only when the modality relationship of the prime and probe was congruent in two consecutive trials, supporting the previous domain-specific view that adjustments in the strength of cognitive control depend on the modality relationship (Yang et al., 2017; Dignath et al., 2019; Nolden and Koch, 2023). Similar findings were also reported by Hazeltine et al. (2011) and Yang et al. (2017) who suggested that the control process is specific to the task set (i.e., the set of rules that define stimulus–response associations) but not to a single stimulus. The control process separately forms visual or auditory task sets on the basis of visual and auditory features and stores these task sets in working memory (Rogers and Monsell, 1995). When these auditory (spoken color words) or visual (characters and letters) stimuli were repeated in the experiment, the participants responded quickly on the basis of the previously stored task sets. These findings highlight the role of congruent contextual features in conflict adaptation.

Notably, few studies have reported significant CSE when two consecutive trials involve the modality switch condition (Hazeltine et al., 2011; Forster and Cho, 2014; Kelber et al., 2024). For example, Grant et al. (2020) investigated the effect of task sets on CSE boundaries and reported that CSE not only appeared in the modality repeat condition but also appeared in the modality switch condition. Additionally, Lee and Cho (2023) used cross-modal Simon tasks to investigate the congruency sequence effects under task switch conditions. The results revealed that a significant CSE was still obtained between the two tasks. As Lim and Cho (2021) indicated, the CSE is transferred across different sensory modalities or tasks because they share the same task-irrelevant stimulus dimension. Furthermore, this modality or task switch evidence might indicate that partial modality repetition across trials might play a general role in CSE (Braem et al., 2014; Grant et al., 2020; Lee and Cho, 2024). For example, in the modality switch condition, a visual–visual trial is followed by a visual–auditory trial, whereas the prime modality feature is the first visual stimulus that represents partial repetition. This partial repetition may enhance the participant’s predictability of the next trial by facilitating modality-specific task set formation (Kreutzfeldt et al., 2016; Lim and Cho, 2018; Grant and Weissman, 2023).

Table 1 shows conflicting results regarding the occurrence of CSE in repeat and switch modalities. For example, Kelber et al. (2023) reported that the CSE occurred only in the repeat modality condition, whereas Grant and Weissman (2023), Exp. 1 reported that the CSE occurred in both the repeat and switch two conditions. Notably, both studies defined repeat modality conditions similarly, but they differed in their definitions of switch conditions. Specifically, the former study defined modality switching as within-trial transitions from visual to auditory (VA), whereas the latter study defined modality switching as between-trial transitions from visual to visual (VV) or auditory to auditory (AA). Thus, such incongruent definitions of modality switch conditions might lead to the formation of distinct task sets (Huber-Huber and Ansorge, 2017; Koob et al., 2023; Siqi-Liu and Egner, 2023). Compared with within-trial transitions, participants are more likely to form task sets during between-trial transitions, which can influence task performance efficiency and accuracy (Schlaghecken and Maylor, 2020; Mackenzie et al., 2022; Brosowsky, 2024). Similarly, for Experiment 1 of Grant et al. (2020), significant CSE was observed only under the repeat condition, whereas significant CSE was observed under both the repeat and switch conditions in Experiment 2, which included two mixed modality transition types: VA-AV and VV-AA conditions. Importantly, such incongruent definitions for the modality switch condition led to these studies not fully excluding partial repetition because response times are faster for full repetitions than for partial repetitions; this feature integration confirms a CSE (Kreutzfeldt et al., 2016; Grant et al., 2022; Bräutigam, 2024). Thus, maintaining the modality relationship under the modality switch condition could avoid the impact of partial repetition to explore whether CSE is specific to the repetition of modalities between trials. However, it remains unclear whether these conflicting findings of modality switching were caused by partial repetition effects under modality transition conditions.

**Table 1:** Definitions of modality transition in cross-modal CSE studies.

Experiment(s)	Task	Transition type		Modality transition		CSE	
		Within	Between	Repeat	Switch	Repeat	Switch
Kelber et al. (2023), Exp. 1	Prime-probe task	Yes		Prime and Probe = AA-AA/VV-VV	Prime and Probe = AV-AV/AV-VA/VA-AV/VA-VA	Yes	No
Grant et al. (2020), Exp. 1	Prime-probe task		Yes	Prime and Probe = AA-AA/VV-VV	Prime and probe = VV-AA/AA-VV	Yes	No
Yang et al. (2017), Exp. 1A,1B	Stimulus response compatibility task	Yes		Location task = AA/VV	Location task = AV/VA	Yes	No
Kreutzfeldt et al. (2016)	Location judgment and Numerical judgment	Yes		Location task and Numerical task = AA/VV	Location task and Numerical task = AV/VA	Yes	No
Hazeltine et al. (2011), Exp. 1	Temporal flanker task		Yes	Flanker and Target = VV/AA	Flanker and Target = VV-AA/AA-VV	Yes	No
Lee and Cho (2023)	Stroop-Simon	Yes		Stroop task and Simon task = AA/VV;	Stroop task and Simon task = VA/AV	Yes	Yes
Grant and Weissman (2019), Exp. 1	Prime-probe task		Yes	Prime and Probe = AA-AA/VV-VV	Prime and probe = VV-AA/AA-VV	Yes	Yes
Grant et al. (2020), Exp. 2	Prime-probe task	Yes	Yes	Prime and Probe = AA-AA/VV-VV	Prime and Probe = AV-AV/AV-VA/VA-AV/VA-VA; Prime and probe = VV-AA/AA-VV	Yes	Yes

A, auditory; V, visual.

The purpose of this study was to investigate whether the CSE is still specific to the modality repeat condition while maintaining the modality relationship of the prime-probe across two consecutive trials, such as VA-VA or AV-AV. Participants are unlikely to group visual and auditory trials into one task set (Lim and Cho, 2018; Grant et al., 2022). Using a cross-modal prime–probe paradigm (Braem et al., 2014; Hazeltine et al., 2011; Kelber et al., 2023), Experiment 1 investigated whether the CSE is domain-specific by maintaining modality relationships and excluding partial repetition. Experiment 1 hypothesizes that CSE might show greater specificity in modality repetition conditions, considering that the evidence suggests enhanced conflict adaptation when contextual features remain constant (Grant and Weissman, 2017; Cracco et al., 2022; Mackenzie et al., 2022). Moreover, in Experiment 1, the repeat condition involved both visual and auditory probe repetitions, which might have introduced modality asymmetry (Bausenhart et al., 2021; Shichel and Goldfarb, 2022; Wang et al., 2012). In prime-probe tasks, the priming stimulus occurring before the probe stimulus may lead to a priming effect. Thus, Experiment 2 aimed to exclude the priming effect to investigate the influence of modality asymmetry on CSE by presenting the prime stimulus and the probe stimulus simultaneously. Experiment 2 tentatively hypothesized that visual priming would trigger a larger cross-modal CSE than would auditory priming, considering visual dominance (Mayer et al., 2016; Spagna et al., 2020; Yu et al., 2022).

## 2 Experiment 1

Experiment 1 aimed to investigate whether the CSE is still specific to the modality repeat condition while maintaining the modality relationship in two consecutive trials. In each trial, the participants had to identify the probe color and ignore the color of the prime. Primes and probes are presented as visual color characters or auditory spoken color words, and the modalities of the prime and the probe are always different. We expected to find a larger CSE when the prime and probe modality repeats (vs. switches) between consecutive trials.

### 2.1 Method

#### 2.1.1 Participants

The sample size for Experiment 1 was determined via G*Power 3.1.9.2 software, indicating that 30 participants were required to detect a medium effect size of *η*^2^ = 0.25 (*α* = 0.05; 1-*β* = 0.90) via a 2 × 2 repeated-measures analysis of variance (ANOVA). Based on an *a priori* power analysis, this is the smallest effect size of the CSE across previous trial congruency (congruent, incongruent) and current trial congruency (congruent, incongruent). It represents the smallest effect size detected in studies examining CSE modulations under various context transitions in prime-probe tasks, as reported in previous research (Hazeltine et al., 2011; Dignath et al., 2019; Grant et al., 2020; Grant and Weissman, 2023).

A group of 39 undergraduate students at Soochow University were enrolled through an experimental recruitment advertisement. Six participants were excluded from the analysis because the data rejection rate exceeded 30%. The final sample consisted of 33 undergraduate students (25 females; *M* = 20.9 years; range = 18–24 years old; SD = 18; 32 right-handed). All participants reported normal or corrected-to-normal vision with no history of neuropsychiatric illness, seizures, or head trauma. All participants provided written informed consent before participating. After the experiment, the participants were paid 20 RMB for their participation. All participants provided their informed consent by completing a consent form that was approved by the Ethics Committee of Soochow University. The study procedures were conducted in accordance with the principles expressed in the Declaration of Helsinki.

#### 2.1.2 Stimuli, tasks and responses

Experiment 1 contained two stimulus types: visual and auditory. The visual stimuli are four Chinese color characters [红, 蓝, 黄, 绿, corresponding to red, blue, yellow, and green in English]. These Chinese color characters are displayed in Microsoft Arial, with a font size of 5 points, presented in the center of the screen, with a visual angle of 2.5°. All visual stimuli are presented on a 23-inch Dell-3020MT display with a resolution of 1920 × 1,080 and a refresh rate of 60 Hz. To create the auditory stimuli, we used Adobe AU software, which converts written text to speech [红(/hong/), 蓝(/lan/), 黄(/huang/), 绿(/lv/)]. The auditory stimuli were presented through a headset earphone (ATH-WS99) at 70 dB.

#### 2.1.3 Design

Experiment 1 consisted of a 2 (modality transition: repeat, switch) × 2 (previous trial congruency: congruent, incongruent) × 2 (current probe modality: visual, auditory) × 2 (current trial congruency: congruent, incongruent) within-subject design. The prime and probe appeared in different sensory modalities, and the prime and probe were presented either auditorily spoken color words or both visually Chinese color characters. There were two consecutive prime–probe pairs, categorized as either modality repeats (e.g., AV–AV or VA–VA) or modality switches (e.g., AV–VA or VA–AV). There are four types of trials (Forster and Cho, 2014; Grant and Weissman, 2017; Kelber et al., 2023): congruent trials following a congruent trial (termed cC), incongruent trials following a congruent trial (termed cI), congruent trials following an incongruent trial (termed iC), and incongruent trials following an incongruent trial (termed iI). In particular, the congruent condition involves a Chinese color character (e.g., 蓝) presented in the prime trial, followed by a semantically congruent probe Chinese speech sound in the probe trial (e.g., /lan/). The incongruent condition refers to a Chinese color character (e.g., 红) presented in the prime trial, followed by a semantically incongruent probe Chinese speech sound in the probe trial (e.g., /lv/).

**Figure 1:**
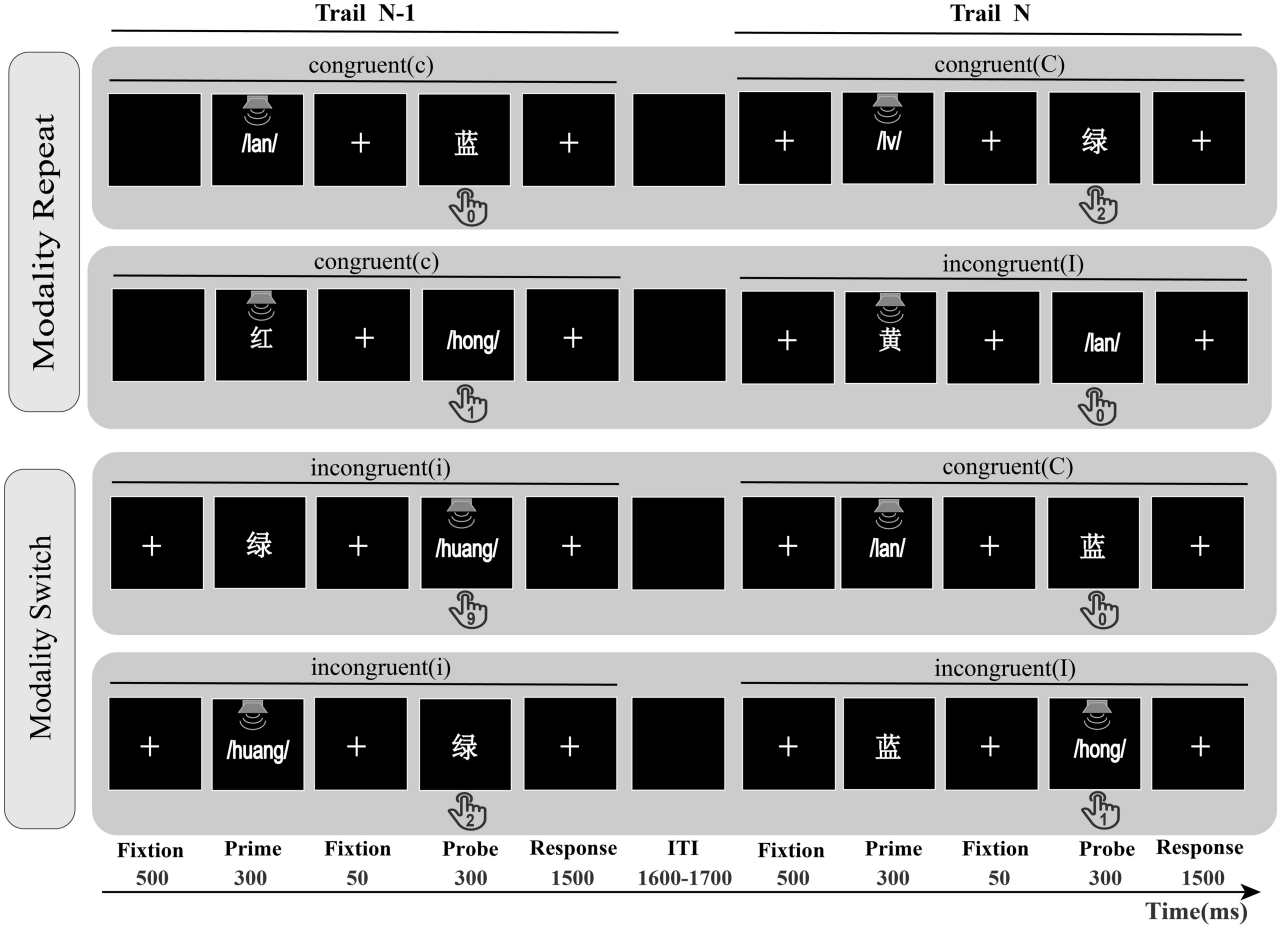
The modality repeat and modality procedures in Experiment 1. Modality repeat: previous probe and current probe are both visual or auditory; Modality switch: previous probe and current probe are different modalities; ITI was randomized from 1,600 ms to 1,700 ms. cC = previous trial congruent, current trial congruent; cI = previous trial congruent, current trial incongruent; iC = previous trial incongruent, current trial congruent; iI = previous trial incongruent, current trial incongruent; Participants had to react to the displayed color words by pressing the corresponding number key on their keyboard.

Figure 1 separately describes two processes of Experiment 1, including modality repeat and modality switch conditions. At the beginning of each trial, a fixation point was displayed in the screen center for a duration of 500 ms, followed by the prime for a 300 ms duration, after a central fixation point for a duration of 50 ms, and finally, a probe appeared for a duration of 300 ms. After the probe offset, the screen remained blank until the participant responded or the time since the probe onset exceeded 1,500 ms. The participants were asked to ignore the initial prime and respond only to the subsequent probe. Specifically, the displayed color words are reacted to by pressing the corresponding number key on their keyboard (the visual stimuli “红, 绿, 黄, 蓝” and the auditory stimuli “/hong/,/lv/,/huang/,/lan/” correspond to the keys “1, 2, 9, 0, respectively”). Following the participant’s response, a blank screen is presented for a duration ranging from 1,600–1,700 ms to minimize the impact of feature integration confounding factors on cognitive control (Polk et al., 2008; Rey-Mermet et al., 2019; Cracco et al., 2022). The experiment presents the stimulus “红/hong/− 绿/lv/” on odd trials and the stimulus “黄/huang/− 蓝/lan/” on even trials (Li et al., 2019; Chen and Zhou, 2013; He et al., 2023); such an approach avoids the repetition of the stimulus–response association in the first and second trials (Gratton et al., 1992; Atalay and Inan, 2017; Kim and Cho, 2014). All visual stimuli were presented on a black background at a viewing distance of approximately 70 cm. The experimental procedure and data recording were controlled by the Psychophysics Toolbox (Brainard and Vision, 1997).

The formal experiment involved 10 blocks presented in a random sequence, and each block included 64 trials lasting 5 min, for a total of 640 trials. The participants could rest for 30 s in each block, and the entire experiment took approximately 50 min to complete. Before the formal experiment began, all the participants were asked to conduct a practice experiment. The practice included 32 trials lasting 2 min; if the participant responded incorrectly or if their reaction time exceeded 1,500 ms, they received feedback on the feedback screen, indicating “Incorrect Response” or “Response Timeout”. The rest of the procedure remains the same as in the formal experiment. The participants were required to achieve an accuracy rate of 80% or higher in the practice sessions to qualify for the formal experiment. Otherwise, additional practice was necessary.

#### 2.1.4 Data analysis

These analyses excluded (1) practice trials, (2) the first trial of each block, (3) error and error posterror attempts in each block, and (4) attempts beyond plus or minus 3 standard deviations at the reaction time from the mean response time (RT) and error rate (ER) data (Grant and Weissman, 2023; Kelber et al., 2023; Moretti et al., 2023). The data from Experiment 1 were organized and subjected to further statistical analysis via MATLAB 2018b and JASP 0.17.1 (Love et al., 2019). The CSE is usually calculated via the following formula (Nieuwenhuis et al., 2006; Atalay and Inan, 2017). CSE = (RT (cI) − RT(cC)) − (RT(iI) − RT(iC)). In this formula, cC, cI, iI, and iC represent four types of conflicts: previous trial congruent and current trial congruent (cC), previous trial congruent and current trial incongruent (cI), previous trial incongruent and current trial congruent (iC), and previous trial incongruent and current trial incongruent (iI).

Four repeated-measures ANOVAs were conducted. First, four-way ANOVAs were separately conducted with modality transition (repeat, switch), previous trial congruency (congruent, incongruent), current probe modality (visual, auditory), and current trial congruency (congruent, incongruent) for RT and ER data. Second, studies have reported that CSE could be modulated by the modality transition between previous and current trials (Hazeltine et al., 2011). Thus, to maintain the consistency of the modality relationship between the prime and probe, the experiment classifies two modality conditions: the current probe repeat condition (VA-VA/AV-AV) and the current probe switch condition (AV-VA/VA-AV), with the aim of identifying differences between the repeat and switch conditions. We conducted a repeated measures analysis of variance (ANOVA) with 2 (modality transition: repeat, switch) × 2 (previous trial congruency: congruent, incongruent) × 2 (current trial congruency: congruent, incongruent) variables.

To further explore modality-specific, two repeated measures analyses of variance (ANOVAs) were performed, 2 (previous trial congruency: congruent, incongruent) × 2 (current trial congruency: congruent, incongruent) factors under trial repetition and switching. Third, the modality repeats are divided into visual probe repetitions and auditory probe repetitions, and two conditions are compared to examine whether the CSE exhibits asymmetry between visual and auditory modalities (Yuval-Greenberg and Deouell, 2009; Donohue et al., 2013; Shaw et al., 2020). Repeated-measures ANOVA was conducted with the current probe modality (visual, auditory), previous trial congruency (congruent, incongruent), and current trial congruency (congruent, incongruent) to investigate the presence of a significant CSE in each specific condition. Specifically, two 2 × 2 repeated measures ANOVAs with previous trial congruency (congruent, incongruent) and current trial congruency (congruent, incongruent) were conducted to identify differences between visual and auditory probes in the CSE. Finally, paired-sample t tests were used to compare the magnitude of CSE (e.g., RT (cI-cC) - RT (iI-iC), Atalay and Inan, 2017; Botvinick, 2007; Gratton et al., 1992) between the visual probe and auditory probe types. Bonferroni correction was performed for the *p* values of the postmultiple comparisons.

### 2.2 Results

The mean response times and error rates are shown in Table 2.

**Table 2:** Average RTs (ms) and ER (%) in Experiment 1 (*M* ± *SD*).

		N congruency	Modality transition(ms)		Modality transition (%)	
Probemodality	N-1 congruency	N congruency	Repeat	Switch	Repeat	Switch
Visual	Congruent	Congruent (cC)	667 ± 111	657 ± 111	3.7 ± 4.4	4.1 ± 3.6
		Incongruent (cI)	748 ± 104	753 ± 100	7.8 ± 6.1	8.3 ± 7.8
	Incongruent	Congruent (iC)	670 ± 101	660 ± 109	3.5 ± 3.5	3.5 ± 4.4
		Incongruent (iI)	722 ± 97	746 ± 99	6.1 ± 5.8	8.5 ± 7.8
Auditory	Congruent	Congruent (cC)	650 ± 122	662 ± 113	3.7 ± 4.7	4.5 ± 3.9
		Incongruent (cI)	806 ± 129	790 ± 114	12.9 ± 10.1	12.3 ± 9.6
	Incongruent	Congruent (iC)	688 ± 110	676 ± 98	5.7 ± 4.7	4.5 ± 8.0
		Incongruent (iI)	763 ± 106	808 ± 120	8.1 ± 7.0	12.3 ± 8.9

RT, response time; ER, error rate; Repeat, the modality relationship between the previous trial and the current trial is congruent; Switch, the modality relationship between the previous trial and the current trial is changed; RT, response time; ER, error rate; cC, congruent trial following another congruent trial; cI, congruent trial following an incongruent trial; iC, incongruent trial following a congruent trial; iI, incongruent trial following another incongruent trial.

#### 2.2.1 Reaction times

The RT and ER results are shown in Table 2. The results revealed significant main effects for the current probe modality, *F* (1, 32) = 12.81, *p* = 0.001, $\eta_{p}^{2}$ = 0.29; current trial congruency, *F* (1, 32) = 93.30, *p* < 0.001, $\eta_{p}^{2}$ = 0.75. Additionally, the results revealed a significant interaction effect between previous trial congruency and current trial congruency, *F* (1, 32) = 34.54, *p* < 0.001, $\eta_{p}^{2}$ = 0.52, and the congruency effect under the previous congruent condition (115 ms) was significantly greater than that under the incongruent condition (86 ms). Significant three-way interactions were also observed. Modality transition, previous trial congruency, and current probe modality had significant interactions, *F* (1, 32) = 8.47, *p* = 0.007, $\eta_{p}^{2}$ = 0.21; the interactions among modality transition, previous trial congruency, and current trial congruency were significant, *F* (1, 32) = 11.78, *p* = 0.002, $\eta_{p}^{2}$ = 0.27. A four-way interaction between modality transition, previous trial congruency, current probe modality, and current trial congruency was significant, *F* (1, 32) = 22.42, *p* < 0.001, $\eta_{p}^{2}$ = 0.41. Importantly, a four-way interaction was found between modality transition, previous trial congruency, current probe modality, and current trial congruency, *F* (1, 32) = 22.42, *p* < 0.001, $\eta_{p}^{2}$ = 0.41.

**Figure 2:**
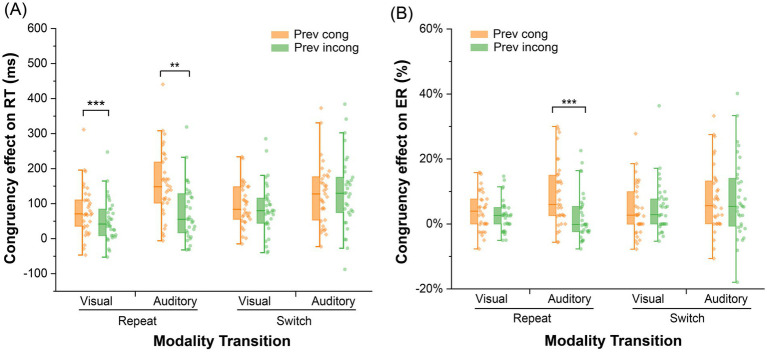
Congruency effects on response time **(A)** and the error rate **(B)** in Experiment 1; congruency effect = RT (incongruent) – RT (congruent); **(A)** congruency effect on response time in Experiment 1; **(B)** congruency effect on the error rate in Experiment 1; Prev cong: previous congruent trial; Prev incong: previous incongruent trial; ***p < 0.001; **p < 0.01; *p < 0.05.

To further explore the significant three-way interaction, we examined whether maintaining consistency in the modality relationship between the prime and probe affects whether the CSE is specific to trial repetition. We conducted two-way ANOVAs with previous trial congruency (congruent, incongruent) and current trial congruency (congruent, incongruent) separately for modality repetitions and switches to investigate whether the CSE was modulated by modality transition. As shown in Figure 2A, for the trial repetition condition, the interaction effect between previous trial congruency and current trial congruency was significant, *F* (1, 32) = 51.63, *p* < 0.001, $\eta_{p}^{2}$ = 0.62, and the congruency effect under the previous congruent condition (119 ms) was significantly greater than that under the incongruent condition (63 ms). Simple effect analysis indicated that, compared with the previous congruent trials, the reaction time after the previous incongruent trials significantly decreased, *t* (32) = 5.71, *p* < 0.001, Cohen’s *d* = 0.33, 95% *CI* = [18.19, 51.43]. The reaction time in the current congruent trials significantly increased, *t* (32) = 3.39, *p* = 0.007, Cohen’s *d* = 0.197, 95% *CI* = [4.07, 37.31]. For the trail switch condition (Figure 2B), there was no significant main effect or interaction effect. In the visual probe switch condition, there was no significant interaction between previous trial congruency and current trial congruency, *F* (1, 32) = 0.16, *p* = 0.69, $\eta_{p}^{2}$ = 0. In the auditory probe switch condition, there was no significant interaction between previous trial congruency and current trial congruency, *F* (1, 32) = 0.83, *p* = 0.37, $\eta_{p}^{2}$ =0.002.

The asymmetry of cross-modal congruency effects was investigated, the differences between visual probe and auditory probe types were compared, and whether the cross-modal congruency sequence effect exhibited asymmetry was explored. Three-way ANOVAs were conducted with the current trial modality (visual, auditory), previous trial congruency (congruent, incongruent), and current trial congruency (congruent, incongruent). There were significant main effects of current trial modality, *F* (1, 32) = 7.55, *p* = 0.01, $\eta_{p}^{2}$ = 0.19; current trial congruency, *F* (1, 32) = 71.24, *p* < 0.001, $\eta_{p}^{2}$ = 0.69. The interaction effect between previous trial congruency and current trial congruency was significant, *F* (1, 32) = 51.63, *p* < 0.001, $\eta_{p}^{2}$ = 0.62, and the congruency effect in the previous congruent condition (119 ms) was significantly greater than that in the incongruent condition (63 ms). The three-way interaction among modality type, previous trial congruency, and current trial congruency was significant, *F* (1, 32) = 22.57, *p* < 0.001, $\eta_{p}^{2}$ = 0.41.

To investigate whether there is a difference in the impact of visual probe and auditory probe modalities on CSE, we conducted two-way ANOVAs with previous trial congruency (congruent, incongruent) and current trial congruency (congruent, incongruent) for the visual probe and auditory probe modalities. For the auditory probe modality, the main effect of current trial congruency was significant, *F* (1, 32) = 73.84, *p* < 0.001, $\eta_{p}^{2}$ = 0.70. The interaction effect between previous trial congruency and current trial congruency was significant, *F* (1, 32) = 73.84, *p* < 0.001, $\eta_{p}^{2}$ = 0.70, and the congruency effect in the previous trial congruent condition (156 ms) was significantly greater than that in the incongruent condition (74 ms). Simple effect analysis indicated that, compared with previous congruent trials, reaction times significantly decreased in the incongruent trials, *t* (32) = 4.92, *p* < 0.001, Cohen’s d = 0.37, 95% *CI* = [19.21, 67.49], and increased in the current congruent trials, *t* (32) = 4.37, *p* < 0.001, Cohen’s *d* = 0.33, 95% *CI* = [14.43, 62.72]. For the visual probe modality, the main effect of current trial congruency was significant, *F* (1, 32) = 39.41, *p* < 0.001, $\eta_{p}^{2}$ = 0.55. The interaction effect between previous trial congruency and current trial congruency was significant, *F* (1, 32) = 9.37, *p* = 0.004, $\eta_{p}^{2}$ = 0.23, and the congruency effect in the previous trial congruent condition (81 ms) was significantly greater than that in the incongruent condition (52 ms). Simple effect analysis revealed that reaction times were significantly lower in the current incongruent trials than in the congruent trials, *t* (32) = 3.84, *p* = 0.002, Cohen’s *d* = 0.25, 95% *CI* = [7.64, 44.91]. However, there was no significant change in reaction times for the current congruent trials (*t* < 1). A paired sample t test was conducted for the CSE of different modalities, and the results revealed that the CSE for the auditory probe type (82 ms) was significantly greater than that for the visual probe type (30 ms), *t* (32) = 3.32, *p* = 0.002, Cohen’s *d* = 0.58, 95% *CI* = [17.26,71.89].

#### 2.2.2 Error rates

The results of the four-way repeated measures ANOVAs revealed a significant main effect of the current probe modality, *F* (1, 32) = 11.17, *p* = 0.002, $\eta_{p}^{2}$ = 0.26; current trail congruency, *F* (1, 32) = 35.57, *p* < 0.001, $\eta_{p}^{2}$ = 0.526. The interaction effect between modality transition and current modality type was significant, *F* (1, 32) = 4.28, *p* = 0.047, $\eta_{p}^{2}$ = 0.118, and the interaction effect between previous trail congruency and current trail congruency was significant, *F* (1, 32) = 5.88, *p* = 0.021, $\eta_{p}^{2}$ = 0.155. The congruence effect in the previous congruent condition (6.3%) was significantly greater than that in the incongruent condition (4.5%). The three-way interaction between trail congruency, current probe modality, and current trail congruency was significant, *F* (1, 32) = 4.24, *p* = 0.048, $\eta_{p}^{2}$ = 0.117. The four-way interaction between modality transition, previous trial congruency, current probe modality, and current trail congruency was significant, *F* (1, 32) = 7.54, *p* = 0.01, $\eta_{p}^{2}$ = 0.191.

To further explain the three-way interaction, two-way ANOVAs were conducted with previous trial congruency (congruent, incongruent) and current trial congruency (congruent, incongruent) for the trail repeat and switch. For the trial repetition condition, the interaction effect between previous trial congruency and current trial congruency was significant, *F* (1, 32) = 12.14, *p* = 0.001, $\eta_{p}^{2}$ = 0.28, and the congruency effect in the previous trial congruent condition (6.6%) was significantly greater than that in the incongruent condition (2.4%). Simple effect analysis revealed that, compared with the previous congruent conditions, the error rate significantly decreased after the incongruent conditions, *t* (32) = 4.13, *p* < 0.001, Cohen’s *d* = 0.68, 95% *CI* = [0.011, 0.056]; the error rate for the current congruent trials showed no significant change. For the trail switch condition, there was no significant main effect or interaction effect. In the visual probe switch condition, there was no significant interaction between previous trial congruency and current trial congruency, *F* (1, 32) = 0.37, *p* = 0.55, $\eta_{p}^{2}$ = 0.002; in the auditory probe switch condition, there was no significant interaction between previous trial congruency and current trial congruency, *F* (1, 32) = 0.002, *p* = 0.97, $\eta_{p}^{2}$ = 0.

To investigate the asymmetry of cross-modal CSE, three-way ANOVAs with current probe modality (visual, auditory), previous trial congruency (congruent, incongruent), and current trial congruency (congruent, incongruent) were conducted. There was a significant main effect of current probe modality, *F* (1, 32) = 9.10, *p* = 0.005, $\eta_{p}^{2}$ = 0.22; current trial congruency, *F* (1, 32) = 35.20, *p* < 0.001, $\eta_{p}^{2}$ = 0.52. The interaction effect between previous trial congruency and current trial congruency was significant, *F* (1, 32) = 11.77, *p* = 0.002, $\eta_{p}^{2}$ = 0.27. The three-way interaction among current probe modality, previous trial congruency, and current trial congruency was significant, *F* (1, 32) = 7.44, *p* = 0.01, $\eta_{p}^{2}$ = 0.19.

To investigate whether there is a difference in the impact of visual and auditory types on CSE, two-way ANOVAs were conducted with previous trial congruency (congruent, incongruent) and current trial congruency (congruent, incongruent). For the auditory probe modality, the interaction effect between previous trial congruency and current trial congruency was significant, *F* (1, 32) = 16.93, *p* < 0.001, $\eta_{p}^{2}$ = 0.35, and the congruency effect was significantly greater in the previous congruent condition (9.1%) than in the incongruent condition (2.3%). Simple effect analysis revealed that, compared with previous congruent trials, the error rate significantly decreased for current incongruent trials following previous incongruent trials, *t* (32) = 4.13, *p* < 0.001, Cohen’s *d* = 0.69, 95% *CI* = [0.02, 0.08], whereas the error rate for current congruent trials remained nonsignificant, *t* (32) = 1.73, *p* = 0.535. For visual probe modality, there was no significant main effect or interaction effect.

### 2.3 Discussion

The purpose of Experiment 1 was to investigate the specificity of CSE by manipulating the congruency of the modality relationship between the prime and probe. The results revealed that the CSE was observed only in the modality repeat condition (Figure 2A) but did not appear in the modality switch condition even when the modality relationship was controlled. These results partially support and extend those of prior related studies, which indicated that CSE was specifically associated with modality repetition when the modality relationship between two trials was congruent (Kreutzfeldt et al., 2016; Lee and Cho, 2024). In this study, the modality repeat was divided into VA and AV repetitions. When comparing the visual and auditory probe conditions, the CSE showed asymmetry between the two modalities (Donohue et al., 2013; Huang et al., 2015; Ulrich et al., 2021; Yu et al., 2022). When the auditory and visual modality types were compared, the CSE of the auditory type was significantly greater than that of the visual type (Figure 2A). This finding provides further support for the existence of a visual advantage in CSE (Bauer et al., 2020; Tomat et al., 2020; Chang et al., 2022).

In Experiment 1, we explicitly controlled the modality relationship between the primes and probes to ensure that, in the switch condition, the relationship was entirely congruent or incongruent. This approach avoids feature integration confounds (Hommel et al., 2001; Huffman et al., 2020; Schiltenwolf et al., 2023) and eliminates partial repetition, which is known to influence cognitive control processes (Bräutigam, 2024). The findings confirmed that CSE is modality-specific and cannot be simply attributed to the repetition of the modality relationship or the formation of visual or auditory task sets. These results align with the theory that task sets, which are defined by context-related features such as sensory modalities, serve as boundaries that guide cognitive control (Grant and Weissman, 2023). The inability of the participants to form a general task set in both the VA and AV switch conditions suggests that modality-specific cognitive control mechanisms are essential for managing cross-modal tasks. Additionally, the results revealed that the CSE was significantly greater under auditory modality conditions than under visual modality conditions (Figure 2A).

The present study extends previous findings of congruent effects on interference within the visual modality (Braem et al., 2019; Jost et al., 2022; Shichel and Goldfarb, 2022) to interference across the visual and auditory modalities. Thus, the recently observed cross-modal flanker effect by Ulrich et al. (2021) can be qualitatively modulated by manipulations similar to visual flanker effects. Not surprisingly, the congruency effects were generally greater with visual distractors than with auditory distractors. Thus, similar to other methods (Frings and Spence, 2010), this finding indicates that visual target processing is more prone to interference within the visual modality than is cross-modal processing (Colavita, 1974; Allenmark et al., 2024). This finding suggests that the visual modality often exerts a stronger influence on cognitive processing than the auditory modality (Ulrich et al., 2021; Li et al., 2023). Moreover, it has been accordingly suggested that vision dominates over audition via enhanced functional connectivity between the dorsal visual stream and the sensorimotor system (Huang et al., 2015; Shaw et al., 2020). This asymmetry may indicate that vision might generally dominate audition, which would lead to greater interference from visual distractors than from auditory distractors. Additionally, the influence of the priming effect also plays a role, as presenting a visual stimulus before an auditory stimulus causes a greater congruency effect than does presenting an auditory stimulus before a visual stimulus (Grant and Weissman, 2017). In Experiment 2, the prime and probe were presented simultaneously to eliminate these priming effects, allowing for a clearer observation of whether modality asymmetry in the CSE still occurs when both modalities are processed concurrently.

## 3 Experiment 2

The purpose of Experiment 2 was to eliminate priming effects to validate CSE differences between visual and auditory probes. In Experiment 2, the prime stimuli were temporally synchronous with the probe stimuli. Before the experiment, the participants were asked to respond selectively to auditory or visual stimuli according to experimental instructions while ignoring another task-irrelevant visual or auditory stimulus. Additionally, the presence of modality asymmetry confirms visual dominance in the VA and AV conditions.

### 3.1 Method

#### 3.1.1 Participants

The sample size for Experiment 2 was determined via G*Power 3.1.9.2 software, indicating that 24 participants were required to detect a medium effect size (*η*^2^ = 0.25) via a 2 × 2 repeated-measures analysis of variance (ANOVA) with a significance level of *α* = 0.05 and a power of 1-*β* = 0.80. A group of 26 undergraduate students from Soochow University were enrolled in an experimental recruitment advertisement. Two participants were excluded from the analysis because the data rejection rate exceeded 30%. The final sample consisted of 24 undergraduate students (21 females; *M* = 21.3 years; range = 18–26 years old; *SD* = 2.2; 24 right- handed). The participants reported normal or corrected-to-normal vision and had no history of neuropsychiatric illness, seizures, or head trauma. All participants also had normal or corrected-to-normal vision and no hearing problems. After the experiment, the participants were paid 20 RMB for their participation. All participants provided their informed consent by completing a consent form that was approved by the Ethics Committee of Soochow University. The study procedures were conducted in accordance with the principles expressed in the Declaration of Helsinki.

#### 3.1.2 Stimuli, tasks and responses

The experimental materials and instruments used are the same as those used in Experiment 1. In particular, the prime stimulus was presented in synchrony with the probe stimuli (Kelber et al., 2023).

#### 3.1.3 Design

**Figure 3:**
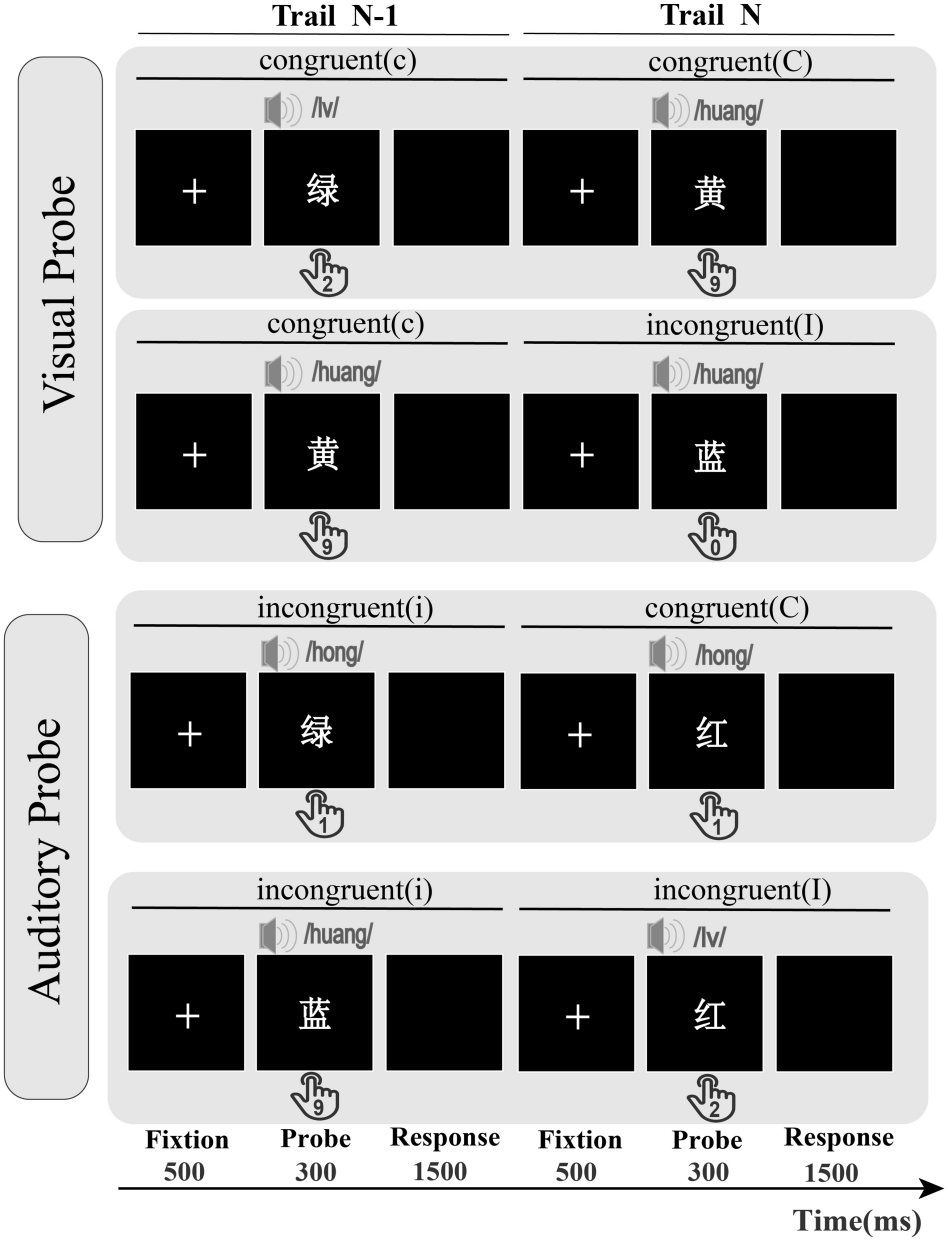
The visual and auditory probe types in Experiment 2; probe modality switches in blocks; cC = previous congruent trial, current congruent trial; cI = previous congruent trial, current incongruent trial; iC = previous incongruent trial, current congruent trial; iI = previous incongruent trial, current incongruent trial; participants had to react to the displayed color words by pressing the corresponding number key on their keyboard.

The experiment employed a cross-modal congruency paradigm in which visual and auditory stimuli were simultaneously presented. The within-subject design with a three-factor structure included the current probe modality (visual, auditory), previous trial congruency (congruent, incongruent), and current trial congruency (congruent, incongruent). The process described above represents the experimental procedure for a single trial of Experiment 2 (Figure 3). Each trial began with a central fixation point displayed (500 ms), followed by a cross-modal stimulus (300 ms), the prime and probe presented at the same time, and then a black screen presented for 1,500 ms until the participants responded. Before each block, the participants were told whether to respond to an auditory probe or a visual probe. The probe modality types alternated between blocks in the ABBA sequence, effectively balancing time order errors into linear system changes (Schneider and Logan, 2006). The keypress requirements matched those of Experiment 1.

The formal experiment consisted of 10 blocks, and one block included 64 trials, for a total of 640 trials. The subjects could rest for 30 s in each block, and the whole task took approximately 40 min to complete. Before the formal experiment began, all the subjects were given a practice experiment. The practice sessions included 32 trials lasting 2 min. The participants received feedback on the feedback screen, labeled “Incorrect Response” or “Response Timeout” in case of errors or response times exceeding 1,500 ms. The participants were required to achieve an accuracy rate of 80% or higher in the practice sessions to qualify for the formal experiment. Otherwise, additional practice was necessary.

#### 3.1.4 Data analysis

In Experiment 2, the data analysis method was the same as that in Experiment 1. To investigate potential differences in CSE between visual probe and auditory probe modality types, a 2 (current probe modality: visual, auditory) × 2 (previous trial congruency: congruent, incongruent) × 2 (current trial congruency: congruent, incongruent) repeated-measures ANOVA was conducted to explore the asymmetry of CSE. Bonferroni correction was performed for the *p* values of the postmultiple comparisons.

### 3.2 Results

The mean reaction time and error rate are shown in Table 3.

**Table 3:** Average RTs (ms) and ER (%) in experiment 1 (M ± SD).

		Probe modality (ms)		Probe modality (%)	
		Visual	Auditory	Visual	Auditory
Congruent	Congruent (cC)	645 ± 139	775 ± 117	6.0 ± 5.2	6.3 ± 5.7
	Incongruent (cI)	673 ± 160	874 ± 112	5.5 ± 5.6	9.8 ± 8.3
Incongruent	Congruent (iC)	648 ± 141	817 ± 123	5.1 ± 5.7	7.0 ± 6.6
	Incongruent (iI)	670 ± 138	873 ± 106	7.4 ± 6.9	7.0 ± 6.1

RT, Reaction time; ER, Error Rate; cC, congruent trial following a congruent trial; cI, congruent trial following an incongruent trial; iC, incongruent trial following a congruent trial; iI, incongruent trial following an incongruent trial.

#### 3.2.1 Reaction time

The RTs and error rates in each condition are shown in Table 3. Three-way ANOVAs with current probe modality (visual, auditory), previous trial congruency (congruent, incongruent), and current trial congruency (congruent, incongruent) were conducted. There were significant main effects of the current probe modality, *F* (1, 23) = 56.58, *p* < 0.001, $\eta_{p}^{2}$ = 0.71; current trial congruency, *F* (1, 23) = 48.71, *p* < 0.001, $\eta_{p}^{2}$ = 0.68. Additionally, the interaction effect between previous trial congruency and current trial congruency was significant, *F* (1, 23) = 11.12, *p* = 0.003, $\eta_{p}^{2}$ = 0.33. The three-way interaction among current probe modality, previous trial congruency, and current trial congruency was significant, *F* (1, 23) = 7.41, *p* = 0.012, $\eta_{p}^{2}$ = 0.24.

**Figure 4:**
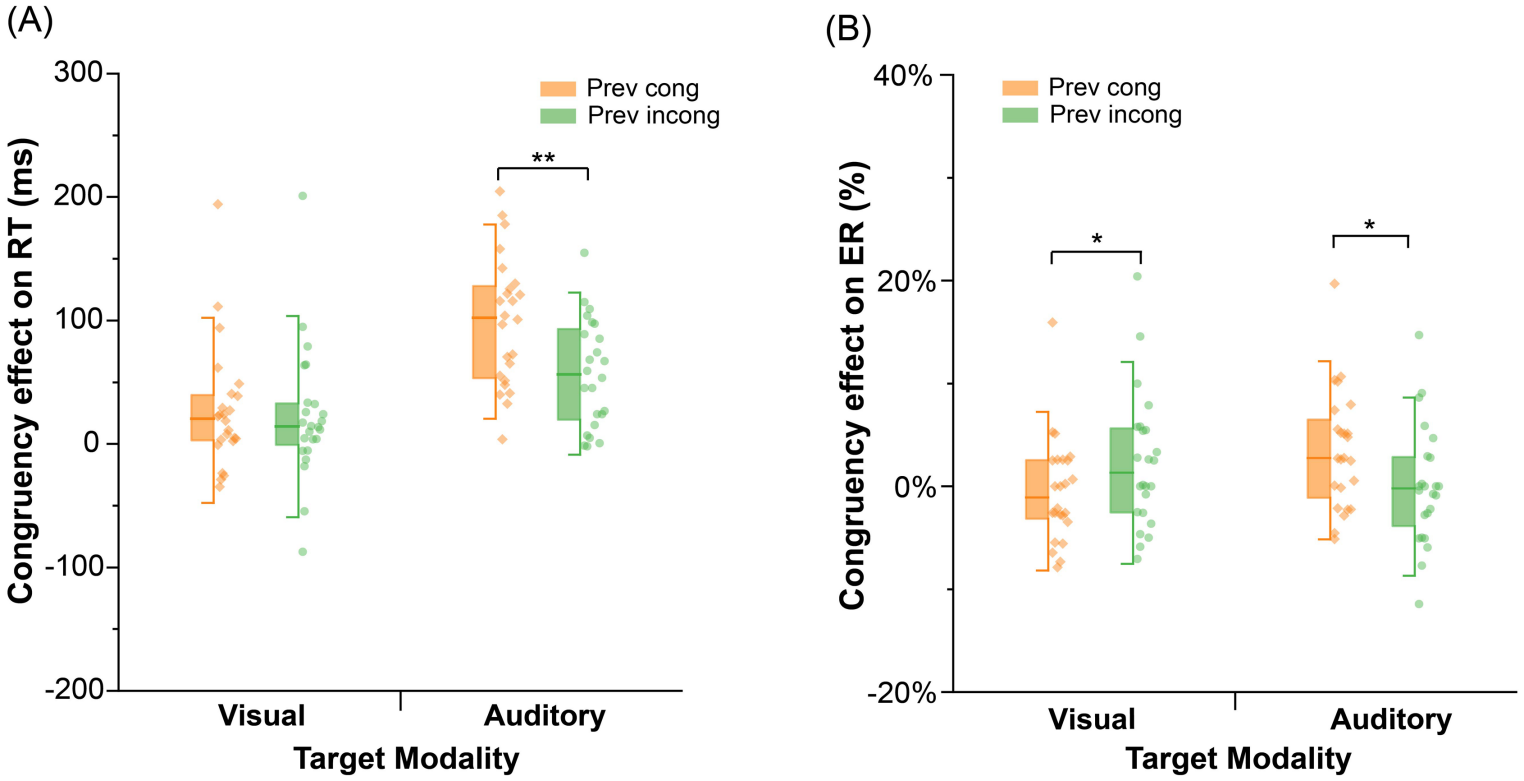
Congruency effects on RT and the error rate in Experiment 2; Congruency effect = RT (incongruent) – RT (congruent); **(A)** congruency effect on RT in Experiment 2; **(B)** congruency effect on the error rate in Experiment 2; Prev cong: previous congruent trial; Prev incong: previous incongruent trial; **p < 0.01; *p < 0.05.

As shown in Figure 4A, two-way ANOVAs were conducted with previous trial congruency (congruent, incongruent) and current trial congruency (congruent, incongruent) among the visual and probe modality types. The auditory probe type had a significant interaction effect between previous trial congruency and current trial congruency, *F* (1, 23) = 31.72, *p* < 0.001, $\eta_{p}^{2}$ = 0.58, and the congruency effect was significantly greater in the previous congruent condition (99 ms) than in the incongruent condition (57 ms). Simple effect analysis revealed that reaction times did not significantly change for current incongruent trials following previous incongruent trials (*t* < 1), whereas reaction times for current congruent trials significantly increased, *t* (23) = 5.36, *p* < 0.001, Cohen’s *d* = 0.36, 95% *CI* = [19.90, 63.16]. For the visual probe type, there was no significant interaction between previous trial congruency and current trial congruency, *F* (1, 23) = 0.19, *p* = 0.67, $\eta_{p}^{2}$ = 0.008.

#### 3.2.2 Error rates

Three-way ANOVAs with current probe modality (visual, auditory), previous trial congruency (congruent, incongruent), and current trial congruency (congruent, incongruent) were conducted on error rates. The three-way interaction among modality type, previous trial congruency, and current trial congruency was significant, *F* (1, 23) = 9.50, *p* = 0.005, $\eta_{p}^{2}$ = 0.29.

As shown in Figure 4B, two-way ANOVAs were conducted with previous trial congruency (congruent, incongruent) and current trial congruency (congruent, incongruent) among the visual and auditory probe modalities. For the auditory probe, the interaction effect between previous trial congruency and current trial congruency was significant, *F* (1, 23) = 5.06, *p* = 0.034, $\eta_{p}^{2}$ = 0.18, and the congruency effect was significantly greater in the previous congruent condition (3.5%) than in the incongruent condition (−0.04%). Simple effect analysis revealed that error rates did not significantly change for current incongruent trials following previous incongruent trials, *t* (23) = 2.04, *p* = 0.316, and error rates for current congruent trials remained nonsignificant (*t* < 1). For the visual probe, the interaction effect between previous trial congruency and current trial congruency was significant, *F* (1, 23) = 4.43, *p* = 0.046, $\eta_{p}^{2}$ = 0.16, and the congruency effect was significantly smaller in the previous incongruent condition (−0.5%) than in the congruent condition (2.3%). Simple effect analysis indicated that error rates did not significantly change for current incongruent trials following previous incongruent trials, *t* (23) = 1.69, *p* = 0.632. The error rates for the current congruent trials remained nonsignificant (*t* < 1).

### 3.3 Discussion

The purpose of Experiment 2 was to validate the differences in the CSE between visual and auditory types by eliminating the priming effects caused by modality asymmetry. The results revealed that modality asymmetry was still observed even after eliminating the priming effect. Specifically, a significant CSE was observed in both the faster reaction time and the lower error rate in the VA condition and not vice versa. The modality asymmetry remained even after presenting the prime and probe stimuli simultaneously to control for priming effects. This finding further indicates that the modality asymmetry in the CSE observed between the VA and AV conditions in Experiment 1 was not entirely caused by the priming effect (Yu et al., 2022; Siqi-Liu and Egner, 2023; Bräutigam, 2024).

The modality asymmetry in the CSE was still obtained even if the priming effect was removed. Experiment 2 suggested that this asymmetry was due mainly to the visual dominance effect in cognitive control processes. The CSE between the previous congruent and incongruent trials in the AV condition was not significant. This suggests that the influence of the previous incongruent trial on the current trial was reduced and may be due to the visual dominance effect (Bauer et al., 2020; Chang et al., 2022; Li et al., 2023). Yu et al. (2022) investigated how the neural encoding of basic auditory and visual features is modulated by cross-modal information when participants watch movie clips that are primarily composed of nonrhythmic events. The results revealed asymmetrical cross-modal interactions in the neural encoding of sensory features. The auditory feature was enhanced by congruent visual information, whereas the neural encoding of visual features was not significantly influenced by auditory input. Thus, in our study, the reduced congruency effect in the AV condition shows that auditory information does not significantly modulate visual processing. This highlights the role of visual dominance in cognitive control processes (Xia et al., 2018; Tomat et al., 2020; Wu et al., 2023). As a result, the cognitive control adjustments triggered by VAs were greater than those triggered by AVs and demonstrated the impact of visual dominance in cross-modal CSE.

## 4 General discussion

After maintaining a consistent modality relationship between the prime and probe across two consecutive trials and excluding the effect of partial repetition, the results of Experiment 1 confirmed that the CSE is indeed specific to cross-trial modality congruency (Kreutzfeldt et al., 2016; Li et al., 2021; Cracco et al., 2022). The findings from Experiment 2 revealed that the CSE was significantly greater under the VA condition than under the AV condition, highlighting the modality-specific effects on cognitive control in cross-modal contexts. The study tentatively suggested that two critical mechanisms might contribute to these results. First, the episodic retrieval view of the CSE might partially explain how memory from previous trials influences cognitive control through the task set. Second, the asymmetry of cognitive control could clarify the visual dominance observed, particularly in the VA and AV conditions.

### 4.1 Episodic retrieval view of the CSE

In Experiment 1, the results revealed significant CSE in the modality repeat condition, which might have led to the formation of modality-specific task sets. This result might be partially supported by the episodic retrieval account provided by Spapé and Hommel (2008), which suggested that participants could create an episodic memory of the previous trial (e.g., the stimuli that appeared) as well as abstract features (e.g., the task, the S–R mapping, trial congruency, Weissman, 2020; Grant et al., 2022). It posits that the CSE is greatest when participants employ the same task set both within each trial and across consecutive trials (Forster and Cho, 2014; Weissman et al., 2016; Dunaway and Weissman, 2024). When the task and/or stimulus modality repeats, the previously formed episodic binding can be reapplied to enhance performance. In the case of a switch of either the task or the stimulus modality, the previous episodic binding must be overcome (Kandalowski et al., 2020).

The current findings suggest that the formation of modality-specific task sets is associated with the orientation of attention toward the relationship between the prime and probe modalities. In our study, we ensured that the modality relationship was fully congruent in the repeat condition and entirely incongruent in the switch condition, preventing participants from predicting the upcoming single-modality task set. Specifically, in the repeat condition, participants categorized each trial on the basis of the probe modality as either “VA” or “AV.” This categorization allows control mechanisms to assign visual and auditory stimuli to distinct tasks, thereby facilitating the formation of modality-specific task sets (Verbruggen et al., 2006; Hazeltine et al., 2011; Koob et al., 2023). Conversely, in the switch condition, the modalities of the previous and current trials were completely incongruent, such as “VA-AV or AV-VA,” making it impossible to establish task sets on the basis of the target modality. Consequently, altering the presentation of task stimuli disrupts the retrieval of episodic memories from prior trials, thereby diminishing or eliminating the CSE. Notably, Grant et al. (2020) employed word stimuli as both prime and probe stimuli in Experiment 2, reporting a significant cross-modal CSE in both repeat and switch modality conditions. Their study included “mixed modality” trials alongside “all visual” and “all auditory” trials, highlighting the critical role of task sets in cognitive control related to the CSE. Importantly, they failed to observe modality-specific CSE, whereas a significant CSE was evident in both the repeat and switch conditions. In contrast, in our study, we observed a significant CSE in the VA and AV repetition conditions. This is in contrast to Grant et al.’s (2020) switch condition, where the VA-AV and VA-VA conditions were treated as switch, without considering that VA-VA is actually a form of VA repetition. We suggest that the modality relationship between prime and probe stimuli may influence the ability of participants to form a task set, leading to a modality-specific CSE.

Additionally, the effects of partial repetition or repetition costs on CSE could also be accounted for by conflict monitoring and cognitive control theories, which posit that the stimuli and their associated responses that occur together are integrated into a single event file. When this pairing is repeated across trials, the episodic memory representation can be readily retrieved, resulting in faster response times (Huffman et al., 2020; Kandalowski et al., 2020). Conversely, when only part of the event file is repeated—such as in instances of partial switching or partial repetition—the nonrepeated portion is automatically activated. This activation must be overridden during the current trial, leading to slower responses. In cases of complete switches, the lack of preactivated representations enables faster responses(Li et al., 2019; Nolden and Koch, 2023). Therefore, in our study, we used four pairs of stimuli with trials consisting of either complete repetitions or complete alternations. For example, the stimuli in trial n-1 could be VA/AV, and in trial n, they could also be VA/AV, representing a complete repetition; alternatively, the stimuli in trial n-1 could be VA/AV, and in trial n, they could be AV/VA, representing a complete alternation. In this way, partial repetition or repetition costs can be completely ruled out as an explanation for the sequential modulation of congruency effects in modality switch trials.

### 4.2 The asymmetry of cognitive control

The results from Experiments 1 and 2 indicated that the CSE was more pronounced in VA conditions than in AV conditions, which suggests a visual dominance effect in cognitive control (Chang et al., 2022; Yu et al., 2022). This can be explained by the fact that visual stimuli tend to dominate cognitive processing across various stages, from early perceptual stages to later stages such as semantic representation and response selection (Thorne et al., 2011; Spence and Ho, 2015). Although the human brain continuously processes inputs from various sensory modalities, it does not assign them equal importance. According to the modality appropriateness hypothesis (Mercier et al., 2015; Wesslein et al., 2019), sensory modalities vary in their suitability for perceiving different types of stimuli. Each modality excels in a specific function, outperforming the others in that area. For example, the visual modality is better suited for detecting object features such as changes in luminance, color, and shape, whereas the auditory modality is more effective in perceiving temporal and frequency aspects of stimuli (Colavita, 1974; Xia et al., 2018). Accordingly, it has been suggested that vision dominates over audition via enhanced functional connectivity between the dorsal visual stream and the sensorimotor system (Huang et al., 2015).

Our findings are partially in line with this evidence, which consistently reported asymmetry between visual and auditory cognitive control processing. Chen and Zhou (2013) reported that visual distractors caused more interference with auditory processing (i.e., typical visual dominance) at the preresponse level, whereas auditory distractors caused more interference with visual processing at the response level. Moreover, Allenmark et al. (2024) demonstrated a reduced priming effect when sensory modalities switch from auditory to visual, where priming was less effective across different modalities than within the same modality. This evidence indicates that modality asymmetry might play an important role in CSE (Botvinick, 2007; Carter et al., 2000; Klatt et al., 2023). Botvinick et al. (2004) offer a framework for understanding visual dominance through the conflict monitoring model; visual information may elicit stronger conflict signals from the dorsolateral prefrontal cortex (DLPFC), which is responsible for conflict resolution by adjusting the weights according to current task demands (Kim and Cho, 2014; Lim and Cho, 2018; Li et al., 2021). Using electrocorticography (ECoG) measurements, Mercier et al. (2015) investigated the role of local and interregional phase alignment in driving a well-established behavioral correlate of multisensory integration. In a speeded detection task, participants responded to auditory or visual and multisensory (audiovisual) stimuli with a button press while electrocorticography was recorded from the auditory cortex. The results showed that visual stimuli reset oscillatory activity in the auditory cortex in the delta (1–4 Hz) and theta bands. This finding supports the idea that a lag between visual and auditory stimuli can influence and reset auditory processing, leading to enhanced visual-to-auditory control mechanisms (Spence and Ho, 2015; Spagna et al., 2020; Sicard et al., 2021). This converging evidence underscores the preferential processing of visual stimuli and explains why the CSE was stronger in VA conditions in our study, highlighting the critical role of visual dominance in cross-modal cognitive control. Furthermore, Xia et al. (2018) investigated the effects of practice on cross-modal selective attention. They focused on whether practice influences different sensory modalities in the same way as the visual dominance observed in multisensory environments. The results demonstrate that practice enhances visual performance, whereas auditory performance remains unchanged, suggesting greater flexibility in adapting visual attention with practice. These findings collectively highlight that the visual modality tends to maintain control across different levels of cognitive processing, as evidenced by the stronger CSE in VA conditions observed in this study.

## 5 Conclusion

The purpose of this study was to explore whether the CSE remains specific to modality repetition. Experiment 1 aimed to maintain consistent modality relationships between trials to eliminate partial repetition effects. The results demonstrated that CSE was significant under repeated conditions, with a stronger effect under VA conditions than under AV conditions. This finding partially supported and extended previous research and suggested an asymmetry in cognitive control between visual and auditory modalities driven by visual dominance. However, Experiment 1 did not address the potential impact of priming effects on cognitive control. Experiment 2 was designed to control for prime effects and further explore the role of modality in the CSE. The results demonstrated that CSE occurred only in the VA condition, which suggested that visual dominance plays a critical role in cross-modal interactions. Overall, the study indicated that the CSE is modality specific, with visual stimuli having a stronger influence on cognitive control than auditory stimuli do. Additionally, the study indicated that the CSE is modality specific, with visual stimuli having a stronger influence on cognitive control than auditory stimuli do. This research contributes to a deeper understanding of cognitive control mechanisms in cross-modal interactions, particularly highlighting the role of visual dominance in shaping modality-specific effects on CSE. In future research, we plan to employ EEG or fMRI techniques to further investigate the neurophysiological mechanisms underlying the CSE. However, as a first step, we aim to thoroughly understand the behavioral mechanisms of the CSE, as this will provide valuable insights for the design of future EEG or fMRI experiments.

## Data Availability

The raw data supporting the conclusions of this article will be made available by the authors, without undue reservation.
